# Psoriasis: To Vaccinate or Not to Vaccinate?

**DOI:** 10.7759/cureus.15860

**Published:** 2021-06-23

**Authors:** Christian Summa, Poonam Patel, Marc M Kesselman, Michelle Demory Beckler

**Affiliations:** 1 Osteopathic Medicine, Dr. Kiran C. Patel College of Osteopathic Medicine, Nova Southeastern University, Davie, USA; 2 Rheumatology, Dr. Kiran C. Patel College of Osteopathic Medicine, Nova Southeastern University, Davie, USA; 3 Immunology, Dr. Kiran C. Patel College of Osteopathic Medicine, Nova Southeastern University, Davie, USA

**Keywords:** immunocompromised patient, psoriasis pathophysiology, psoriasis treatment, vaccination, vaccinations' role in psoriasis

## Abstract

Psoriasis is a chronic, inflammatory, autoimmune disease characterized by red, dry, itchy, and scaly patches of abnormal skin growth on the elbows, knees, and/or scalp, which can negatively impact a patient’s quality of life and activities of daily living. Both genetic predispositions and environmental factors, which can vary in susceptibility and effect, including infection, stress, medications, and cold temperatures, can lead to the onset of psoriasis and progression of the condition. This review aims to highlight recent advances in understanding the pathophysiology of psoriasis and provide insight into the importance of vaccinations and their role in reducing the risk of infection in psoriasis patients. Vaccination has been shown to reduce the risk of infection in psoriasis patients and those with other autoimmune diseases.^ ^Still, vaccination remains limited among autoimmune disease patients.^ ^Awareness of the benefits of vaccination needs to be raised among healthcare professionals due to the overarching impact on these patients’ lives. The focus of this literature review is to examine the existing data to determine whether vaccination is beneficial for psoriasis patients. Herein, we primarily focus on influenza, pneumococcal, and herpes zoster vaccines and whether immunization benefits or adversely affects psoriasis patients. Overall, we found that most psoriasis and vaccine literature support immunization of this patient population, particularly with non-live attenuated vaccines; however, more studies are needed to fully develop a vaccine recommendation schedule for psoriasis patients.

## Introduction and background

Psoriasis is a chronic, inflammatory, autoimmune disease that has been shown to negatively impact an individual’s quality of life, doubling ideation about suicide [1]. Data has demonstrated a genetic predisposition and non-specific environmental factors, including infection, stress, medications, and cold temperatures, can lead to the onset and progression of the condition [2].

Psoriasis can develop in various forms, including plaque, inverse, guttate, and pustular psoriasis, each having similar and distinct clinical phenotypes [3]. Nearly all psoriasis forms present with lesions that have common characteristics, ranging in size from a pin to 20 cm in diameter. The most common form of psoriasis is plaque psoriasis, which presents with elevated, red skin lesions that are covered with silver scales, which can arise anywhere on the body. Inverse psoriasis often occurs within skinfolds, such as the armpits, groin area, or under the breasts. This condition presents with bright, red, smooth lesions that flare up due to heat and sweat. These lesions can also be found on the extremities as small, scaly, and shaped like water droplets. Guttate psoriasis typically occurs among young adults and children and presents repeatedly. This condition arises after bacterial infections, such as *Streptococcus*. Pustular psoriasis is the least common form of psoriasis and presents with red lesions with pus and other symptoms, including fever, chills, fatigue, and/or nausea [2].

The pathogenesis of psoriasis has yet to be fully elucidated. Research efforts have highlighted that the onset and progression of psoriasis are likely associated with a genetic predisposition, including HLA-Cw*0602 alleles and the presence of autoantigens triggered by an environmental factor. In addition, other immune factors play a role in disease activity, including T helper 17 (TH 17) cells, interleukin (IL)-17, and IL-23, which have been implicated in the positive pro-inflammatory feedback loop that leads to hyperproliferation of keratinocytes, ultimately leading to the physical presentation of the disease via skin lesions. In addition, IL-17/IL-23 and T cells appear to be the major contributors to the innate and adaptive immune responses underlying the cyclical epidermal inflammatory episodes seen in the condition [4]. The levels of these cytokines, along with others derived from TH 17 cells, in skin lesions as well as in peripheral blood have been shown to be elevated in psoriasis patients as compared to their healthy counterparts [5]. IL-17 has also been shown to act on dermal fibroblasts in stromal tissue leading to the production of pro-inflammatory cytokines that promote the expression of keratin 17, which is the only keratin that has been found in the lesions of psoriasis patients [6].

## Review

Infection and vaccination

As researchers and clinical practitioners gain a better understanding of the pathophysiology of psoriasis, treatments have become more targeted toward the underlying mechanisms of disease activity. Targeted treatments, including biologics, aim to provide a more effective and safer approach to psoriasis treatment because they specifically target dysregulated immune mechanisms rather than non-specifically suppressing the entire immune system [7]. Despite the success, these treatments leave the patient immunologically susceptible to infection. Additionally, recent publications suggest that psoriasis is not just a disease of the skin but a disease of systemic inflammation. Thus, psoriasis patients are at an increased risk for infection, most commonly infections in the lower respiratory tract, particularly pneumonia [4]. Despite these patients' enhanced susceptibility, vaccination rates remain low among psoriasis patients [8,9]. Potential explanations for low vaccination rates include concerns about the safety and efficacy of the vaccination and patients being unaware of an increased risk for infection tied to biologic therapy and the illness itself [10]. To address this issue, below, we highlight available studies on vaccination in psoriasis patients. To determine whether vaccination is beneficial, we performed a comprehensive review of the literature using PubMed, Ebsco, and Medline databases. Key search terms used were: psoriasis, psoriatic, vaccination, vaccine, immunization, influenza vaccine, pneumococcal, herpes zoster, measles, mumps, rubella, BCG, hepatitis, etc.

Influenza

Psoriasis patients have been shown to have a higher risk of contracting influenza compared to the general population [11]. Seasonal influenza is an infectious disease with high mortality rates, especially among immune-compromised and autoimmune disease patients. Still, many psoriasis patients decline to obtain the vaccine, as noted above [12]. Contributing to the low rates, there has been some suggestion that the influenza vaccine can increase levels of cytokines associated with epidermal changes and/or lead to psoriatic flare-ups. H1N1 influenza vaccination did appear to have the potential to trigger psoriatic flare-ups; however, there seems to be a low incidence and a mild clinical course [2,13]. Ultimately, there is a lack of evidence to support this connection. Despite the need for further research, the influenza vaccination has been shown to provide promising results based on current data. Radtke et al. showed that 28 percent of psoriasis patients in their study accepted the seasonal vaccine; of these, only 1.08 percent of vaccinated patients contracted influenza showing the efficacy of the vaccine in preventing influenza in psoriasis patients [12]. 

Pneumonia

Pneumonia is a major health threat in autoimmune disease patients, especially those treated with immunosuppressive therapy. For example, long-term use of methotrexate in rheumatoid arthritis patients increases the relative risk of developing pneumonia 9.7 times in non-vaccinated compared to vaccinated patients [11]. Furthermore, anti-tumor necrosis factor (TNF) therapy (adalimumab, infliximab, or etanercept) can decrease the strength of patients’ humoral and cell-mediated immunity, leaving them susceptible to *Streptococcus pneumoniae* infections [14]. A recent study showed that patients treated with infliximab, specifically, had the highest risk of infections as an adverse effect, leading to discontinuation of this immunosuppressive drug therapy [15]. Thus, the correlation between immunosuppressive therapy and pneumococcal susceptibility has been established.

Establishing protection against infection is the mainstay purpose of vaccinations. The pneumococcal vaccine is a polysaccharide vaccine composed of bacterial cell wall components. Lynde et al. assessed antibody titer levels in plaque psoriasis patients after the 23-valent pneumococcal polysaccharide vaccination, Pneumovax-23 (Merck, Kenilworth, USA) [16]. This study showed that 86 percent of patients had a two-fold or greater increase in antibody titers from pre-vaccination to six weeks post-vaccination and 57 percent of vaccinated patients had a four-fold increase in antibody titers or better at six weeks, suggesting a protective immune response in response to vaccination. This study also examined the effect of vaccination on psoriasis flare-ups and found no evidence of an increase in flare-ups as a result of pneumococcal vaccination [16]. Moreover, three patients exhibited clearance of all psoriatic symptoms after vaccination [16]. 

Overall, these preliminary studies show that pneumococcal vaccination is beneficial to psoriasis patients and has little to no adverse effect. More studies are needed to further solidify this conclusion. In addition, in the future, it will be important to study the efficacy of Prevnar 13 (Pfizer, Groton, USA), another pneumococcal vaccine, in psoriasis patients. 

Herpes Zoster

In the US, every year, there are more than one million cases of herpes zoster (HZ) virus infection [17]. One subset of patients particularly affected is the elderly. Due to HZ prevalence in the elderly community, there is a question as to whether the incidence rate is high enough to warrant vaccinations at a younger age for immunosuppressed patients especially. Additionally, studies have shown that the frequencies of HZ events are several times higher among patients with autoimmune or inflammatory diseases [18]. Notedly, patients currently treated with glucocorticoids, methotrexate, tofacitinib, or other biological agents have a higher HZ incidence rate [17,19]. A recent study concluded that patients treated with tofacitinib have an increased risk for HZ, two to three-fold higher than patients on TNF alpha inhibitors [5].

The newest vaccine, Shingrix (GlaxoSmithKline Biologicals, Middlesex, United Kingdom), is an inactivated subunit vaccine that contains a single strain of inactivated shingles virus. Shingrix was approved for use in 2017 and has been shown to have a 97 percent efficacy. Meanwhile, no data is available regarding efficacy specifically in psoriasis patients [20].

Studies have demonstrated that Zostavax (Merck & Co., Inc., Kenilworth, USA) vaccination of psoriasis patients is beneficial at protecting patients from contracting HZ [17]. Yun et al. showed that psoriasis patients vaccinated with HZ live attenuated vaccine [Zostavax; recommended in patients over 60 years or older by the U.S. Centers for Disease Control and Prevention (U.S. CDC)] were less likely to experience HZ incidence than those who were not vaccinated [18]. Similarly, Zhang et al. showed that only 0.15 percent of psoriasis patients that were vaccinated developed HZ events, and only one patient in this study developed primary varicella [17]. In addition, a large cohort study of Medicare patients was conducted whereby the live attenuated HZ vaccine was administered to patients over 60 years old. The results, post vaccination day 42 status, reported no increase in the incidence of HZ in the vaccinated individuals. Combined, these studies strongly indicate that the HZ vaccine provides sufficient benefit for this cohort of patients. 

Bacillus Calmette-Geurin

Immunizations appear to be relatively effective in psoriasis patients. Still, there were two case reports where individuals developed the condition with no known history of the condition. In the first case, a 60-year-old patient with no prior family history of psoriasis was diagnosed with high-grade urothelial carcinoma of the bladder and treated with six weekly installments of the Bacillus Calmette-Geurin (BCG) immunotherapy. The BCG vaccination is a live attenuated *Mycobacterium bovis* vaccine that is primarily used to prevent severe forms of *Mycobacterium tuberculosis* infection, but it is also used to treat bladder cancer [21]. Two months post-treatment, this patient reported multiple skin lesions on his trunk, back, and extremities and was ultimately diagnosed with guttate psoriasis. Three weeks after the patient underwent dermatological therapy; he resumed the BCG immunotherapy, which, in turn, caused a psoriatic relapse [21]. In the second case, a six-month-old developed psoriatic lesions that were hypothesized to have resulted from the BCG vaccine. One month post-vaccination, plaques initially developed at the site of injection, followed by multiple plaques on the face and extremities. The authors hypothesize that the immunological reaction to the BCG vaccination may mediate the production of IL-22 producing TH 17 cells and lead to the activation of epidermal Stat-3, causing a psoriatic skin reaction [22]. More studies are needed to confirm this correlation between the BCG vaccine and psoriatic lesions. 

Discussion and recommendations 

In the most recent recommendations (Table 1), non-live/inactivated vaccines can be safely administered to psoriasis patients regardless of the underlying therapy. This includes hepatitis A, hepatitis B, influenza, pneumococcal, tetanus toxoid, and human papillomavirus (HPV), though our literature searches revealed efficacy and safety studies only for influenza and pneumococcal vaccines. However, the usage of live attenuated vaccinations is more complex. As per the new guidelines, influenza and pneumococcal vaccination are recommended among patients with psoriasis (Table 1). Tetanus and HPV vaccination should be administered similarly to the general population (Table 1); however, this is not discussed in this review article because our searches did not reveal any substantial studies on these vaccinations in psoriasis patients. Hepatitis A, B, and herpes zoster vaccines are suggested to be administered among higher-risk populations (Table 1). Additional studies are needed to improve upon current vaccination guidelines specific to psoriasis patients. Based on the limited knowledge to date, it is preferred for patients to receive vaccinations during the quiescent phase of their disease. In patients with active disease, immunization should be considered on an individual basis. 

**Table 1:** Live Attenuated and Inactivated Vaccinations Recommended for Psoriasis Patients BCG and yellow fever vaccinations are not recommended. Although tetanus toxoid was not addressed in this paper, tetanus toxoid vaccination recommendation coincides with the general public. Additionally, hepatitis A vaccine has been recommended to at-risk populations, including those traveling to or residing in endemic countries. The second dose is recommended to those that are on immunosuppressive therapies. Hepatitis B vaccine is recommended to patients at risk, including those traveling, patients at increased risk of exposure, such as IV drug users, men having sex with men, and needle stick exposure.

Live Attenuated	Inactivated
Measles​, mumps, and rubella (MMR)	Influenza vaccine
Varicella-zoster	Pneumococcal
Herpes zoster	Meningococcal
Influenza	Hepatitis A
	Hepatitis B

For live attenuated vaccinations, it is recommended that these vaccines are administered four weeks prior to the initiation of treatment. However, the exception to this rule includes measles, mumps, and rubella (MMR) and herpes zoster vaccines. An anecdotal study suggests that the MMR and booster vaccine should be considered for administration to psoriasis patients with low-grade immunosuppression [23]. U.S. CDC defines low-grade immunosuppression as a person using glucocorticoids greater than or equal to two weeks in a dosage equivalent to prednisone 20 mg/d or 2mg/kg body weight.

## Conclusions

Often, moderate to severe psoriasis patients are treated with highly beneficial immunosuppressive therapies. Despite the success, these treatments leave the patient immunologically susceptible to infection. Vaccination has become a proven strategy at reducing this risk of infection, despite vaccination rates remaining low among these patients. Physicians should be aware of the positive impact vaccinations can have on immunosuppressed patients, reducing the risk of morbidity and mortality.

For moderate to severe psoriasis patients, the recommendation is to have an assessment of their immunization status, including history for *Haemophilus influenza*, *Tetanus, Pertussis*, *Varicella-zoster*, hepatitis A and B, human papillomavirus, influenza, and *Streptococcus pneumoniae*, preferably before any treatment regimen is commenced, especially for those patients prescribed methotrexate, a strong immunosuppressant. While there is good evidence that patients with psoriasis would immunologically benefit from influenza, pneumococcal, and HZ vaccinations, further studies are needed on other vaccinations.

## References

[1] Liang SE, Cohen JM, Ho RS (2019). Psoriasis and suicidality: a review of the literature. Dermatol Ther.

[2] Gunes AT, Fetil E, Akarsu S, Ozbagcivan O, Babayeva L (2015). Possible triggering effect of influenza vaccination on psoriasis. J Immunol Res.

[3] Nickoloff BJ, Wrone-Smith T (1999). Injection of pre-psoriatic skin with CD4+ T cells induces psoriasis. Am J Pathol.

[4] Hawkes JE, Yan BY, Chan TC, Krueger JG (2018). Discovery of the IL-23/IL-17 signaling pathway and the treatment of psoriasis. J Immunol.

[5] Baumrin E, Van Voorhees A, Garg A, Feldman SR, Merola JF (2019). A systematic review of herpes zoster incidence and consensus recommendations on vaccination in adult patients on systemic therapy for psoriasis or psoriatic arthritis: from the medical board of the National Psoriasis Foundation. J Am Acad Dermatol.

[6] Joost I, Steinfurt J, Meyer PT, Kern WV, Rieg S (2016). Staphylococcus aureus bacteremia with iliac artery endarteritis in a patient receiving ustekinumab. BMC Infect Dis.

[7] Graves JE, Nunley K, Heffernan MP (2007). Off-label uses of biologics in dermatology: rituximab, omalizumab, infliximab, etanercept, adalimumab, efalizumab, and alefacept (part 2 of 2). J Am Acad Dermatol.

[8] Lowes MA, Suárez-Fariñas M, Krueger JG (2014). Immunology of psoriasis. Annu Rev Immunol.

[9] Munz OH, Sela S, Baker BS, Griffiths CE, Powles AV, Fry L (2010). Evidence for the presence of bacteria in the blood of psoriasis patients. Arch Dermatol Res.

[10] Rahier JF, Moutschen M, Van Gompel A (2010). Vaccinations in patients with immune-mediated inflammatory diseases. Rheumatology (Oxford).

[11] Pithadia DJ, Reynolds KA, Lee EB, Wu JJ (2019). Translating the 2019 AAD-NPF guidelines of care for the management of psoriasis with biologics to clinical practice. Cutis.

[12] Radtke MA, Rustenbach SJ, Reusch M, Strömer K, Augustin M (2013). Influenza vaccination rate among patients with moderate to severe psoriasis. J Dtsch Dermatol Ges.

[13] Sbidian E, Eftekahri P, Viguier M (2014). National survey of psoriasis flares after 2009 monovalent H1N1/seasonal vaccines. Dermatology.

[14] Ovejero-Benito MC, Prieto-Pérez R, Llamas-Velasco M (2017). Polymorphisms associated with etanercept response in moderate-to-severe plaque psoriasis. Pharmacogenomics.

[15] Sivamani RK, Correa G, Ono Y, Bowen MP, Raychaudhuri SP, Maverakis E (2010). Biological therapy of psoriasis. Indian J Dermatol.

[16] Lynde C, Krell J, Korman N, Mathes B (2011). Immune response to pneumococcal polysaccharide vaccine in adults with chronic plaque psoriasis treated with alefacept. J Am Acad Dermatol.

[17] Zhang J, Xie F, Delzell E (2012). Association between vaccination for herpes zoster and risk of herpes zoster infection among older patients with selected immune-mediated diseases. JAMA.

[18] Yun H, Yang S, Chen L (2016). Risk of herpes zoster in autoimmune and inflammatory diseases: implications for vaccination. Arthritis Rheumatol.

[19] Winthrop KL, Curtis JR, Lindsey S (2017). Herpes zoster and tofacitinib: clinical outcomes and the risk of concomitant therapy. Arthritis Rheumatol.

[20] Cunningham AL (2016). The herpes zoster subunit vaccine. Expert Opin Biol Ther.

[21] Hung CT, Wang WM, Tsao CW, Chiang CP (2012). New-onset guttate psoriasis following intravesical immunotherapy of Bacillus Calmette-Guerin. Dermatol Sin.

[22] Takayama K, Satoh T, Hayashi M, Yokozeki H (2008). Psoriatic skin lesions induced by BCG vaccination. Acta Derm Venereol.

[23] Worth A, Waldman RA, Dieckhaus K, Rothe MJ (2020). Art of prevention: our approach to the measles-mumps-rubella vaccine in adult patients vaccinated against measles before 1968 on biologic therapy for the treatment of psoriasis. Int J Womens Dermatol.

